# Naphthoquinone glycosides for bioelectroanalytical enumeration of the faecal indicator *Escherichia coli*

**DOI:** 10.1111/1751-7915.12373

**Published:** 2016-07-01

**Authors:** Jamie Hinks, Evelina J. Y. Han, Victor B. Wang, Thomas W. Seviour, Enrico Marsili, Joachim S. C. Loo, Stefan Wuertz

**Affiliations:** ^1^ Singapore Centre for Environmental Life Sciences Engineering (SCELSE)Nanyang Technological University60 Nanyang DriveSingapore637551; ^2^ School of Materials Science and EngineeringNanyang Technological UniversitySingapore639798; ^3^ School of Civil and Environmental EngineeringNanyang Technological UniversitySingapore639798; ^4^ Department of Civil and Environmental EngineeringUniversity of California, DavisOne Shields AvenueDavisCA95616USA

## Abstract

Microbial water quality monitoring for the presence of faecal indicator bacteria (FIB) is a mandatory activity in many countries and is key in public health protection. Despite technological advances and a need for methodological improvements, chromogenic and fluorogenic enzymatic techniques remain the mainstays of water quality monitoring for both public health agencies and regulated utilities. We demonstrated that bioelectroanalytical approaches to FIB enumeration are possible and can be achieved using commercially available enzyme‐specific resorufin glycosides, although these are expensive, not widely available or designed for purpose. Following this, we designed two naphthoquinone glycosides which performed better, achieving *Escherichia coli* detection in the range 5.0 × 10^2^ to 5.0 × 10^5^ CFU ml^−1^ 22–54% quicker than commercially available resorufin glycosides. The molecular design of the naphthoquinone glycosides requires fewer synthetic steps allowing them to be produced for as little as US$50 per kg. Tests with environmental samples demonstrated the low tendency for abiotic interference and that, despite specificity being maintained between β‐glucuronidase and β‐galactosidase, accurate enumeration of *E. coli* in environmental samples necessitates development of a selective medium. In comparison to a commercially available detection method, which has U.S. Environmental Protection Agency (EPA) approval, our approach performed better at high organism concentrations, detecting 500 organisms in 9 h compared with 13.5 h for the commercial method. Bioelectroanalytical detection is comparable to current approved methods and with further development could result in improved detection times. A recent trend for low‐cost open‐source hardware means that automated, potentiostatically controlled *E. coli* detection systems could be constructed for less than US$100 per channel.

## Introduction

There is a link between exposure to human waste and the transmission of infectious diseases like cholera, typhoid fever, shigellosis and acute gastroenteritis (Lee *et al*., 2002; Ashbolt, 2004; U.S. EPA, 2012). Considerable effort is expended to detect, quantify and track faecal contamination of water to ensure drinking water quality meets sanitary guidelines (Cabral, 2010; Nshimyimana *et al*., 2014) and that recreational waters remain safe for primary and secondary contact activities such as swimming and boating. Usually, faecal indicator bacteria (FIB) are monitored as a proxy for pathogenic faecal contamination of water in conjunction with risk assessment techniques that correlate the frequency of a specific hazard (such as the occurrence of gastroenteritis) with a given level of FIB exposure.

FIB monitoring commonly involves enumerating total coliforms (TC) and faecal coliforms (FC), also referred to as thermotolerant coliforms (TTC), which includes *Escherichia coli*. Faecal enterococci are the preferred indicator for marine waters in the United States (U.S. EPA, 2012) and have more recently been listed as admissible for freshwater alongside *E. coli* (U.S. EPA, 2012). Alternative indicators, such as *Clostridium perfringens* and *Bacillus* spp., have been used as FIB in an attempt to definitively identify human faecal contamination of water (Kemmochi *et al*., 2007; Douterelo *et al*., 2014). In addition, cultivation‐independent methods targeting the order *Bacteroidales* have used 16S rRNA‐based genetic markers with faecal host‐associated distribution and geographic stability (Seurinck *et al*., 2005; Wuertz *et al*., 2011; Liang *et al*., 2012; Reischer *et al*., 2013; Odagiri *et al*., 2015). Similarly, immunological tests like ELISA exist, but have not been routinely applied (Wang *et al*., 2007).

Regulatory resistance to emerging FIB enumeration technologies remains, even though the range of indicator proxies and potential enumeration technologies has improved (Lazcka *et al*. 2007). Consequently, few regulatory guidelines exist prescribing standard protocols for molecular approaches to FIB monitoring. An exception is the U.S. EPA guidelines permitting the application of qPCR for enumerating *Enterococcus faecalis* in recreational water quality monitoring. Despite drawbacks, such as their indirect nature, time required for growth and assumptions underlying FIB selection, FIB still remain important tools in assessing microbial water quality. Along with traditional culture techniques, enzymatic enumeration of FIB is favoured for evaluating microbial water quality under most regulated jurisdictions (Douterelo *et al*., 2014; TMR, 2015).

Bioelectroanalytical approaches are emerging as a method of scrutinizing microbial systems (Seviour *et al*., 2015). Although specialist microbes can reduce electrodes directly, it is a near universal property of microbes to indirectly reduce an electrode via soluble redox mediators (Rabaey *et al*., 2005, 2007; Mathis *et al*., 2008). Although more commonly applied for biocatalysis (Rabaey and Rozendal, 2010) or electricity production (Logan and Regan, 2006), cell–electrode interactions can be used to qualitatively or quantitatively describe features of living microbial systems by monitoring the redox processes they mediate. Furthermore, the advent of low cost, open source and electrochemical components, such as the CheapStat potentiostat, has enabled inexpensive hand‐held, multi‐analyte detection systems to be developed, reportedly for as little as US$30 (Rowe *et al*., 2011; Nemiroski *et al*., 2014). With regards to analysis of environmental samples, there are additional benefits to bioelectroanalytics, including increased durability and less interference from deeply coloured or fluorescent matrices (Nijak *et al*. 2012; Hata *et al*. 2014; Kapoor *et al*. 2015). Furthermore, bioelectronalytical systems are inherently scalable, a property which has been extensively discussed in the literature in the context of microbial fuel cells (Logan, 2009a,b).

**Figure 1:**
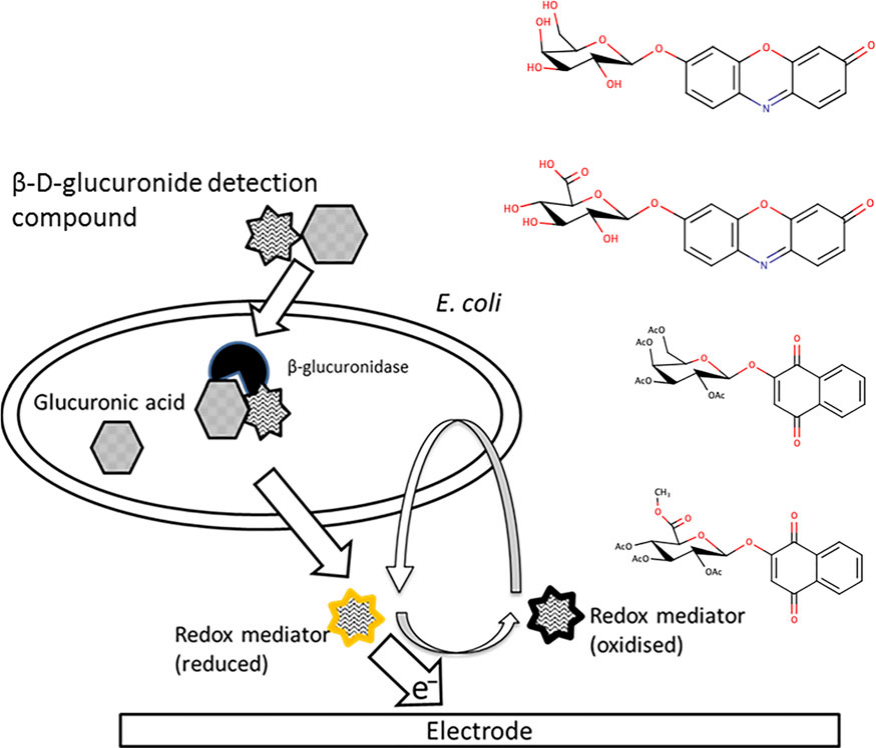
Mechanism of bioelectroanalytical detection technique and the detection compounds used here (from top to bottom): resorufin‐β‐d‐galactopyranoiside; resorufin‐β‐d‐glucuronide and novel peracetylated methyl‐esters of 2‐hydroxy‐1,4‐napthoquinone‐β‐d‐galactopyranoside and 2‐hydroxy‐1,4‐napthoquinone‐β‐d‐glucuronide.

Enzymatic detection of FIB usually involves a β‐glucuronidase‐ or β‐galctosidase*‐*specific monosaccharide conjugated to a chromogenic or fluorogenic compound (aglycon). When cleaved by the corresponding enzyme, the aglycon is released into the medium and detected optically and the presence of the target organism is inferred (Manafi *et al*., 1991; Nelis and Van Poucke, 2000). Because *E. coli* is not strongly electrogenic, incorporating redox‐active aglycons into the enzymatic substrate would enable targeted *E. coli* bioelectroanalysis and would represent a natural extension to the chemistry underlying optical techniques. Such an approach would find utility in bioelectroanalytical approaches to FIB enumeration while simultaneously satisfying industrial and regulatory preference for enzymatic techniques. Previous electrochemical attempts at *E. coli* enumeration have combined techniques to separate the medium from the growing culture for analysis in a separate electrochemical procedure (Perez *et al*., 2001). Perez *et al*. (2001) achieved electrochemical FIB detection using 4‐aminophenol‐β‐D‐galactopyranoside. However, with their experimental set‐up, pre‐filtration and flow injection analysis (FIA) were required. We contend that specifically designed electrochemically active glycosides, which mediate the reversible transfer of electrons between *E. coli* and an electrode, are key to bioelectranalytical approaches (Fig. 1). By exploiting the natural redox cycling capabilities of target microbes, bioelectrochemical detection of *E. coli* can be achieved without sample preparation in a single pot analysis for the majority of environmental samples.

Resorufin can be reversibly reduced and oxidized by microorganisms and is used as a redox indicator in anaerobic microbiology (Candeias *et al*., 1998). Resorufin glycosides make excellent candidates for bioelectroanalytical *E. coli* enumeration, although this has not been previously demonstrated. Resorufin glycosides are commercially available but because resorufin is not available in bulk quantities, it is expensive as are its derivatives (Magro *et al*., 2014). The aim of this study is to demonstrate that the rational design of redox‐active glycosides can be applied to achieve performance benefits in electroanalytical enumeration of *E. coli* over resorufin glycosides. We present two novel naphthoquinone glycosides specifically designed for the bioelectroanalytical enumeration of FIB. The candidate aglycons are analogues of the redox component of menaquinone, a naturally occurring, membrane‐bound charge carrier in *E. coli* that can be readily conjugated to pyranose rings (Unden, 1988; Qiao *et al*., 2008). Here, we demonstrate the feasibility of the bioelectranalytcial approach using resorufin glycosides, we expound on the development of naphthoquinone glycosides, demonstrate their improved performance and finally we explore the potential utility of redox‐active glycosides in the eventual bioelectroanalytical detection of FIB. To our knowledge, this is the first study that considers FIB‐specific glycosides that have been specifically selected and rationally designed for bioelectroanalytical enumeration.

## Materials and methods

### Microorganism and growth conditions

*Escherichia coli* strain BL21 was selected to represent a FIB of human origin that contains the *uidA* and *lacZ* genes, i.e. a TTC. *Aeromonas hydrophila* (DSM 6173), on the other hand, was chosen as an environmental organism that has the *lacZ* gene but which is not considered a coliform. All organisms were cultured aerobically for 20 h. *E. coli* was grown in LB broth (Lennox) (37°C), and *A. hydrophila* in tryptone soy broth at 30°C in shaking incubator. Overnight cultures were diluted in sterile‐modified M9 medium [Na_2_HPO_4_·7H_2_O (6.8 g l^−1^); KH_2_PO_4_ (3.2 g l^−1^); NH_4_Cl (1 g l^−1^); NaCl (0.5 g l^−1^)]; 1 ml l^−1^ vitamin stock solution, 0.2 ml l^−1^ micronutrients stock solution (Mathews *et al*., 2010); tryptone (12.5 g l^−1^); HEPES (6 g l^−1^). The carbon source was either D‐galacturonic acid or D‐glucuronic acid (3.75 g l^−1^) to achieve final inoculum densities in the range of 1.0 × 10^0^, 5.0 × 10^0^, 5.0 × 10^1^, 5.0 × 10^2^, 5.0 × 10^3^, 5.0 × 10^4^ and 5.0 × 10^5^ CFU ml^−1^.

### Mediators and glycosides

Resorufin (TCI‐Europe, Zwijndrecht, Belgium), 2‐hydroxy‐1,4‐naphthoquinone (2H14NQ; TCI‐Europe), 5‐hydroxy‐1,4‐naphthoquinone (5H14NQ; TCI‐Europe) and 5,8‐dihydroxy‐1,4‐naphthoquinone (58H14NQ; Sigma‐Aldrich, Singapore) were obtained commercially as were resorufin glycosides (resorufin‐β‐d‐galactopyranoside (ResGal) and resorufin‐β‐d‐glucuronide (ResGlu); Sigma‐Aldrich), whereas 2‐hydroxy‐1,4‐naphthoquinone‐β‐d‐galactopyranoside (2H14NQGal) and 2‐hydroxy‐1,4‐naphthoquinone‐β‐d‐glucuronide (2H14NQGlu) were custom synthesized by Sussex Research (Ottawa, ON, Canada). All unconjugated mediators and conjugated glycosides were added to achieve a final concentration of 50 μM. Stock concentrations (1 mM) of 2H14NQ and 5H14NQ were prepared by first dissolving in 10 μl of 2 M NaOH and then making up to volume in ultrapure water (Veolia, Paris, France), whereas 58H14NQ (1 mM) was prepared in 1:1 DMSO:ultrapure water aliquots. Both stock solutions were stored at −20°C until needed. Glycosides were maintained as 1 mM stock solutions in ultrapure water and stored at 5°C.

### Environmental samples

Three environmental samples were collected from a private lake on Nayang Technological University campus on March 10th (Day 1), 17th (Day 2) and 25th (Day 3), 2016. A 1000 ml grab sample was collected from the bank. The water temperature was consistently 33°C, whereas the air temperature ranged between 31.5 and 36.5°C.

The sample taken on Day 1 was filter sterilized and stored at 4°C until use, at which point it was warmed and spiked with 5 × 10^3^ CFU ml^−1^ of *E. coli* BL21 prior to duplicate bioelectranalytical analysis at 37°C with ResGlu to detect *E. coli* only. The samples taken on Day 2 were used for TC and *E. coli* enumeration, and were analysed immediately after transport to the laboratory using both the bioelectroanalytical approach (in duplicate) and Colifast^®^ Quanti‐Tray 2000^®^ according to the manufacturer's instructions. On Day 3, the bioelectroanalytical approach was used to enumerate TC only. Incubation for both methods was at 37°C. For the enumeration using bioelectroanalytical approach, autoclaved 10× modified M9 (as described above) was added to environmental water samples to achieve a working volume of 10 ml and a glycoside concentration of 50 μM. TC were estimated from non‐weighted exponential regression of the data presented in Table 1 for ResGal at 37°C. The standard curve, the regression coefficient and the equation are presented in Fig. S1. As the detection times observed for *E. coli* were out of the range of the standard curve, no estimation of CFUs in environmental samples was made.

**Table 1:** Mean enumeration times for *Escherichia coli* with inoculum sizes of 5.0 × 10^2^, 5.0 × 10^3^, 5.0 × 10^4^ and 5.0 × 10^5^ CFU ml^−1^ achieved with two commercially available glycosides (ResGlu and ResGal) and two compounds described for the first time in this study (2H14NQGal and 2H14NQGlu). Detection time is calculated from chronoamperometry performed in ECs and expressed as the mean detection time in minutes. *n* = 3 except * where *n* = 1

Cell count (CFU ml^−1^)	*E. coli* enumeration time (h)					
	ResGal (30°C)	2H14NQGlu (30°C)	ResGal (37°C)	ResGlu (37°C)	2H14NQGal (37°C)	2H14NQGlu (37°C)
5 × 10^2^	8.6 (2.03)^a^	13.9*	12.2 (3.13)	7.2 (0.50)	6.9 (0.23)	5.7 (0.70)
5 × 10^3^	6.2 (1.38)	9.6*	7.6 (0.38)	6.6 (0.88)	5.9 (0.22)	3.1 (0.53)
5 × 10^4^	3.6 (0.52)	n/d^b^	5.1 (0.92)	5.2 (0.56)	2.4 (0.29)	2.4 (0.20)
5 × 10^5^	2.5 (0.93)	2.8*	4.3 (0.92)	4.3 (0.32)	1.8 (0.14)	2.0 (0.35)

a One standard deviation in parentheses.

b Not determined.

### Construction of electrochemical cells

Conical ECs with a working volume of 10 ml and equipped with carbon‐felt electrodes were used. The ECs were fitted with a customized Teflon cap to accommodate a three‐electrode configuration and gas fittings. The carbon‐felt working electrodes (1 × 1 × 0.318 cm; VWR, Singapore), pre‐treated for 24 h in 1 M HCl and stored in deionized water until use, were connected to titanium wire (0.25 mm diameter; Sigma‐Aldrich) using a nylon nut and bolt. A coiled titanium wire counter electrode and an Ag/AgCl reference electrode (78 mm × 6 mm outer diameter; Biologic, Claix, France) were connected using a 1 M KCl in 1.5% agar salt bridge ending in a 4 mm glass frit (CoralPor^™^; Biologic). The entire assembly except the Ag/AgCl reference electrode and 1.5% 1 M KCl salt bridge was autoclaved (121°C for 15 min). Nine millilitres of degassed modified M9 medium were added to the ECs and supplemented with mediator to a final concentration of 50 μM and inoculated with the test organism to achieve a final working volume of 10 ml. The headspace was flushed continuously with sterile, humidified N_2_ to maintain a moist anaerobic atmosphere. The ECs were operated at 30, 37 or 44.5°C in the dark to prevent photo‐bleaching of the light‐sensitive mediators. Stirring was maintained continuously with a magnetic stirrer.

### Electrochemical data acquisition

Electrochemical cells were connected to a multichannel potentiostat (Biologic), and cyclic voltammetry (CV) and differential pulse voltammetry (DPV) were performed immediately but without data collection for diagnostic purposes and to discharge the electrode. Data were recorded by EC‐Lab software (Biologic). Chronoamperometry (CA) was applied for up to 24 h to detect the activity of *E. coli* via redox mediators. A detection event was calculated when the slope of the line exceeded four times the standard deviation of the baseline and detection times are expressed as the mean of triplicate analyses unless stated otherwise. A sample raw electrochemical output can be seen in Fig. S2.

The parameters for the electrochemical techniques were chosen accordingly: CA: E_applied_ = 200 mV versus Ag/AgCl; CV: equilibrium time of 5 s; scan rate of 10 mV/s; E_i_ = −700 mV versus Ag/AgCl; E_f_ = 500 mV versus Ag/AgCl; DPV: E_i_ = −700 mV versus Ag/AgCl; E_f_ = 500 mV versus Ag/AgCl; pulse height of 50 mV; pulse width of 200 ms; step height of 2 mV; step time of 400 ms and scan rate of 5 mV/s.

## Results and discussion

### Bioelectroanalytical enumeration with resorufin glycosides

Enumeration of *E. coli* was achieved with resorufin‐β‐d‐galactopyranoside (ResGal) at 37°C in ECs in 4–12 h for inoculum sizes ranging between 5.0 × 10^5^ and 5.0 × 10^2^ CFU ml^−1^ with good reproducibility across triplicate analysis (Table 1). For resorufin‐β‐d‐glucuronide (ResGlu), enumeration time was achieved more quickly at 37°C (between 4.3 and 7.2 h) for a similar inoculum size (Table 1). Abiotic controls show the absence of current (Fig. S3) and the detection time seems to correlate well with the time when cultures enter log phase (Fig. S4).

**Figure 2:**
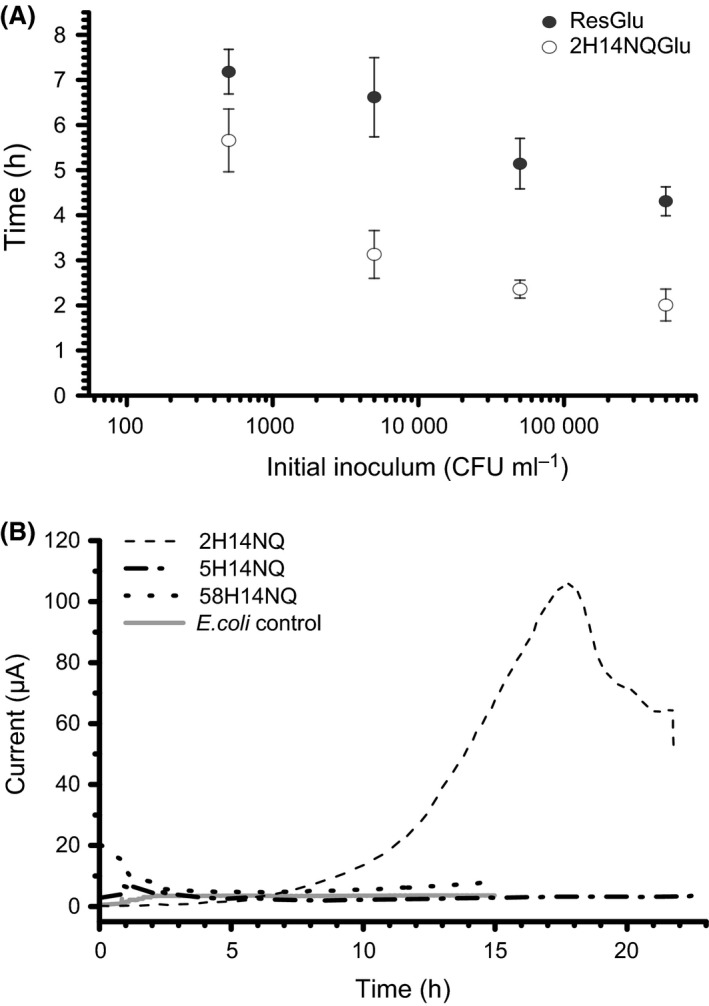
A. Detection time for different inoculum sizes of *Escherichia coli* incubated at 37°C with a commercially available detection compound, ResGlu, and also the novel 2H14NQGlu designed in this study (error bars indicate standard deviation, *n* = 3). B. Duplicate current profiles of candidate redox‐active aglycons in ECs. Note that 2H14NQ outperforms the other candidate compounds (5H14NQ and 58H14NQ) in terms of onset time and maximum signal intensity, which is sustained over longer periods, despite only minor differences in structure. Note the absence of significant current in the controls incubated with *E. coli* only.

Electrochemical enumeration of *E. coli* using chromogenic glycosides has been reported previously (Perez *et al*., 2001), where an enumeration time of 6.5 h was documented for an inoculum size of 5.0 × 10^3^ CFU ml^−1^ *E. coli* in samples not induced with isopropyl‐β‐d‐1‐thiogalactopyranoside. This was faster than the enumeration time of 7.6 h reported here for ResGal at 37°C (Table 1), but comparable with the time of 6.6 h that was achieved for ResGlu at 37°C (Fig. 2A).

Enumeration times for colorimetric β‐glucuronidase assays have been reported to be as low as 0.5 h with high sensitivity (1 CFU ml^−1^), but these techniques require several pre‐treatment steps, such as filtration, incubation and staining in addition to specialist detection equipment. The time saving is offset by a concomitant increase in required manpower or complexity, effectively making these approaches labour intensive and laboratory based (Fiksdal *et al*., 1994; Nelis and Van Poucke, 2000; Heery *et al*., 2016). Whereas the sensitivity of these rapid techniques is undeniably high (detecting 4‐APGal in quantities as low as 45 ng l^−1^), the quantification step usually requires alkaline conditions to maximize the optical properties of the aglycon, further complicating the procedure although they have utility in field detection particularly when FIB concentrations are expected to be high. Despite these advances, the detection time for market‐leading, U.S. EPA approved enzymatic *E. coli* detection methods is in the range 15–28 h for drinking water applications (TMR, 2015, Colifast, 2016, Idexx, 2016).

Similarly, the method reported by Perez *et al*. (2001) required a filtration step and, in addition, a combination of FIA and a potentiostatic technique to achieve electrochemical detection of 4‐aminophenyl (4‐AP) that had been cleaved by *E. coli* from 4‐aminophenyl‐β‐d‐galactopyranoside (4‐APGal) glycoconjugate. The complexity and low throughput of this method make it less amenable to automated or remote applications when compared with the documented durability and low cost of bioelectroanalytical methods that do not require pre‐treatment and which could be combined with automated sampling devices (Logan, 2009a,b; Nemiroski *et al*., 2014). Furthermore, 4‐APGal and 4‐nitrophenol‐β‐d‐glucuronidase (4‐NPglu) have been designed for colorimetric and fluorimetric assays. Although they have some electrochemical activity, their redox chemistry is not fully reversible by *E. coli* under physiological conditions the redox kinetics are slow. Because *E. coli* is not strongly electrogenic in character (Roller *et al*., 1984; Choi *et al*., 2003; Wang *et al*., 2013), the utility of non‐reversible chromogenic substrates in bioelectroanalytical methods is restricted to techniques, such as voltammetry, that do not rely on or fully exploit microbially mediated electron transfer that is achievable in bioelectroanalytical systems.

Low‐cost bioelectroanalytical methods would be amenable to automation and portable applications. The approach presented here is unsophisticated and unoptimized using low‐cost carbon‐felt electrodes to directly measure an actively respiring culture without pre‐treatment. The scope for refinement and to improve enumeration times remains considerable assuming that the cost of the electrode can be offset by the design of cost‐effective glycosides.

### Experimental rationale for napthoquinone glycosides

Although ResGal can be used to quantify *E. coli*, it is not available in bulk and is, therefore, expensive (Biosynth, 2012, Magro *et al*., 2014). Our source cost more than US$ 3000 per gram and export records suggest prices in excess of US$ 1000 per kg are typical for resorufin (Zauba, 2016). Naphthoquinones are cheap, readily available redox‐active molecules, which are compatible with *E. coli* (Park and Zeikus, 2000; Rau *et al*., 2002). Because naphthoquinones possesses a hydroxyl group, they can be readily conjugated to pyranose rings (Magro *et al*., 2014). Their ability to mediate electron transfer between *E. coli* and an electrode in ECs shows evidence of their potential as candidate redox‐active *E. coli* detection compounds (Fig. 2B). Furthermore, they can be acquired in bulk for as little as US$ 50 per kg making the raw materials 20 times cheaper than resorufin (Zauba, 2016).

Glycoside synthesis requires that the carboxyl and hydroxyl groups of the sugar be protected during synthesis with a methyl and an acetyl group, respectively. Deprotection yields can be as low as 5% (Shen *et al*., 2003; Biosynth, 2012). Peracetylated methyl‐esters conjugated to cheap redox mediators make good candidate glycosides and may offset the cost of the electrode in bioelectroanalytical *E. coli* detection. Synthesis of the resulting glycoside would require no deprotection step, resulting in increased yields and therefore reduced production costs compared with fully deprotected glycosides. Because *E. coli* cells express native esterase, they are biochemically equipped to convert the peracetylated methyl‐esters into the acid form *in situ* if this is necessary for subsequent electrochemical activity (Antonczak *et al*., 2009). If the deprotection step, with yields between 5% and 50%, is omitted, then naphthoquinone peracatelyated methyl‐ester glycosides could be as much as 40–800 times cheaper than resorufin glycosides (Biosynth, 2012). Magro *et al*. (2014) estimate the cost of chromogenic glycosides to be in the range US$ 500–1000 per kg. The naphthoquinones are in the same price range as chromogenic substrates leading to a cost estimate for naphthoquinone glycosides in the range US$ 50–500 kg. On a per test basis this could be as little as 0.005 cents assuming on current concentrations of 50 μM (Biosynth, 2012, Magro *et al*., 2014; Zauba, 2016).

Of three candidate naphthoquinones: 2‐hydroxy‐1,4‐naphthoquinone (2H14NQ), 5‐hydroxy‐1,4‐naphthoquinone (5H14NQ) and 5,8‐dihydroxy‐1,4‐naphthoquinone (58H14NQ) tested, 2H14NQ outperformed both 5H14NQ and 58H14NQ in ECs (Fig. 2B). The maximum recorded current in ECs incubated with 5.0 × 10^5^ CFU ml^−1^ *E. coli* and using 2H14NQ as the redox mediator was ~100 μA (Fig. 2B), which was ~14 times the maximum current achieved with 5H14NQ (~7 μA) and ~25 times that observed with 58H14NQ (~4 μA). Accordingly, 2H14NQ was selected over 5H14NQ and 58H14NQ for conjugation to both galacturonic acid and glucuronic acid on the basis of its sound electrochemical performance and its potential to enable targeted bioelectroanalytical enumeration of *E. coli*. It has been previously reported that electron transfer kinetics are dependent on the stability of intramolecular hydrogen bonds of the various naphthoquinones (Frontana and González, 2005). There are two types of hydrogen bond systems in the naphthoquinones studied here: 2H14NQ has an α‐hydroxy system (where the interaction between the carbonyl group and the hydroxy group is supported on the same ring), whereas 5H14NQ and 58H14NQ have β‐hydroxy systems (where the intramolecular interaction occurs between adjacent rings). Electron transfer kinetics is affected by the β‐hydroxy systems and likely underlies the poor bioelectrochemical performance of 5H14NQ and 58H14NQ. The suspected incompatibility of β‐hydroxy systems in bioelectroanalytical *E. coli* enumeration is interesting and offers valuable insights into the future selection and design of candidate glycosides (Frontana and González, 2005).

### Performance of novel glycosides

Both 2H14NQGal and 2H14NQGlu can be used to enumerate *E. coli*, confirming sound rationale in molecule selection. The enumeration time for *E. coli* using 2H14NQGlu ranges between 2 and 5.7 h (±7.7–16.6%, *n* = 3) for inoculum sizes between 5.0 × 10^5^ and 5.0 × 10^2^ CFU ml^−1^ (Fig. 2A). The enumeration time achieved with 2H14NQGlu is quicker than that observed for ResGlu (4.3–7.2 h) (Table 1) and compares well with the enumeration time of ~6.5 h for 5.0 × 10^3^ CFU ml^−1^ reported by Perez *et al*. (2001). However, the value reported by Perez *et al*. (2001) was for a galactopyranoside conjugate. The galactopyranoside equivalent (2H14NQGal) used here resulted in only a slightly slower enumeration time of ~6.9 h (±3.3%, *n* = 3) versus 6.5 h reported by Perez *et al*. (2001) for an equivalent inoculum size. In this study, the naphthoquinone glycosides achieved faster enumeration times than their resorufin counterparts, demonstrating that peracetelylated methyl‐ester glycosides offer both cost savings and performance benefits in the context of bioelectroanalytical *E. coli* detection. Variations in glycoconjugate performance have previously been attributed to transport phenomena, particularly cellular uptake (Magro *et al*., 2014). It may be, therefore, that the relatively small size of the naphthoquinone glycoconjugate compared with the resorufin glycosides allow for improved transport across the cell membrane and, hence, better performance.

### Applicability of bioelectroanalytical approach to environmental TC detection

A remote biosensor would ideally have as few components as possible. We explored the possibility of omitting the heating component of a detection unit and if TC monitoring at ambient Singapore temperatures (~30°C) was achievable. The inoculum density of *E. coli* correlated well with the electrochemical response of ResGal at 30°C with delays in enumeration time of up to 60%. The enumeration times with 2H14NQGlu were more negatively impacted by temperature (Table 1). Even though detection at ambient temperatures is possible, faster detection times could be realized with incubation at 37 or 44.5°C.

Environmental samples may present a number of challenges for bioelectralanytical detection, particularly abiotic interference from redox‐active compounds and the possibility of interference from non‐target bacteria. To rule out the importance of abiotic interference, a filter sterilized lake water sample was spiked with a known number of *E. coli* and analysed using the bioelectroanalytical technique with 2HN14QGlu. The detection time was longer than expected and, by extrapolation from the data in Table 1 for ResGal at 37°C, a sample of 5 × 10^3^ CFU ml^−1^ was estimated to contain < 500 CFU ml^−1^ (Table 2). This likely reflects the documented loss of viability observed in laboratory strains when introduced to environmental samples rather than electrochemical interference, which would be expected to speed up the analysis time (Bissonnette *et al*., 1975). The natural environmental concentration of compounds that could cause interference such as humics is typically over 100 times lower (< 0.22 mg l^−1^) than the redox mediator added here (≈ 24 mg l^−1^) and are not likely to interfere with the analysis (Vuorenmaa *et al*., 2006).

**Table 2:** The analysis time for a filter sterilized environmental sample spiked with known number of *Escherichia coli* and a comparison of a colorimetric reference method with the proposed bioelectroanalytical approach. Total coliforms and *E. coli* were estimated in environmental water samples collected over three different days. Bile salts were included in one treatment on day 3 to demonstrate the need for selective media. For the reference method, *n* = 1, whereas for the bioelctroanalytical method *n* = 2, ±1 S.D in parentheses

Method	Time to detection (h)	Total coliforms (CFU ml^−1^)	*E. coli* (CFU ml^−1^)
Day 1			
Bioelectroanalytical (spiked)	8.46 (±0.54)	n/a	< 500^b^
Day 2			
Quanti‐Tray 2000	24	36.5	0.031
Bioelectroanalytical	7.43 (± 0.36)	1030^a^ (± 374)	< 500^b^
Day 3			
Quanti‐Tray 2000	24	92.8	0.26
Bioelectroanalytical	5.47 (± 0.10)	3628^a^ (± 620)	n/a
Bioelectroanalytical (with bile salts)	7.27 (± 0)	< 500^b^ (± 0)	n/a

a Detection time calculated from standard curve for ResGal (Fig. S4).

b Where the detection time lays outside the calibration range, the value has been reported as < 500 CFU.

Enzymatic methods typically exploit the *uidA* gene, which encodes for the β‐glucuronidase enzyme, and is found in almost 98% of *E. coli* (Manafi *et al*., 1991; Martins *et al*., 1993). Similarly, the *lacZ* gene, which encodes for β‐galactosidase, is indicative of TC such as *Klebsiella* spp., *Citrobacter* spp. as well as *E. coli* (Bej *et al*., 1991; Manafi *et al*., 1991; Cabral, 2010). However, although conferring some degree of specificity, enzymatic tests in themselves are not definitively differential because a number of non‐target organisms are positive for either β‐glucuronidase or β‐galactosidase. In samples, particularly of environmental origin, that are expected to contain a variety of organisms, measures that prevent the growth of non‐target organisms accompany enzymatic tests, such as the inclusion of a selective medium and or elevated incubation temperatures (often 44.5°C). In addition, agents that help in the recovery of environmentally stressed cells such as pyruvate are added to the medium to improve performance (Kapuscinski and Mitchell 1981; Khaengraeng and Reed 2005). Organisms known to contribute to false positives in β‐glucuronidase and β‐galactosidase enzymatic tests include members from the genera: *Staphylococus*,* Sphingomonas*,* Bacillus*,* Aerococcus*,* Entrobacter*,* Pseudomonas* and, notably, *Aeromonas* which is often considered to be a nuisance in *E. coli* monitoring.

Tests with environmental samples confirmed interference from non‐target organisms. When compared with a standard reference method there was a 30‐fold overestimation of TC using our approach (1030 CFU ml^−1^) compared with the reference method (36.54 CFU ml^−1^) and an almost 200‐fold difference in *E. coli* estimation (5.84 CFU ml^−1^ versus 0.031 CFU ml^−1^). This points to the lack of selective agents in our approach compared with the reference method and that, although a degree of selectivity between galactosidase and glucuronidase positive organisms has been maintained, the accuracy is low due to inability to discriminate between target and non‐target organisms. To demonstrate the importance of a selective medium, we ran additional tests with and without bile salts. In this test, the reference method estimated TC as 92 CFU ml^−1^, whereas the bioelectroanlaytical approach with and without bile salts estimated the concentration as 3660 (±620) and 408 (±0) CFU ml^−1^, respectively (Table 2). Bile salts are commonly used to inhibit some non‐target microbes demonstrating the importance of selective agents for the accurate estimation of *E. coli* in environmental samples.

**Figure 3:**
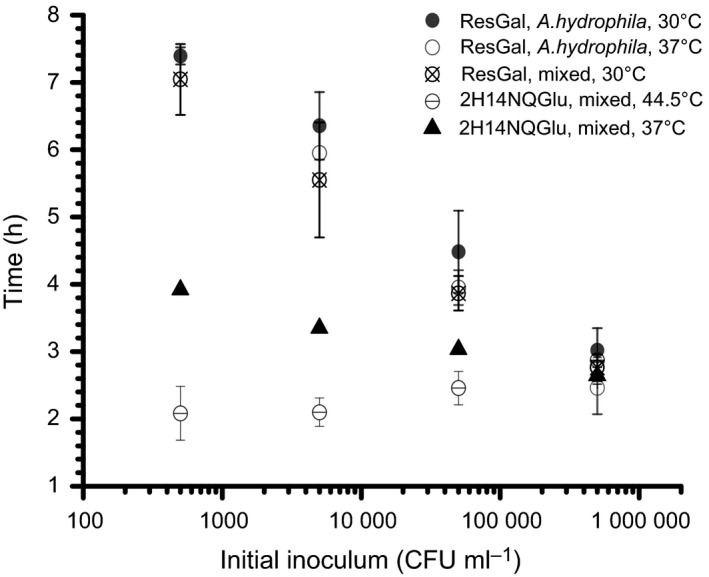
Detection time of a pure culture of *Aeromonas hydrophila* at 30 and 37°C as a function of inoculum size (filled and unfilled circles, respectively), and of a mixed culture of *A. hydrophila* and *Escherichia coli* at 30°C as a function of *A. hydrophila* inoculum size but with a constant *E. coli* inoculum of 5.0 × 10^5^ CFU ml^−1^ (crossed empty circles). The glycoside is the commercially available ResGal. In addition, the detection time for a mixed culture of *A. hydrophila* and *E. coli* at 37°C (black triangles) and 44.5°C (crossed circles) as a function of *A. hydrophila* inoculum size is included. The *E. coli* inoculum is kept constant at 5.0 × 10^5^ CFU ml^−1^ and the glycoside is the novel 2H14NQGlu (error bars represent 1 standard deviation, *n* = 3).

The ubiquitous aquatic organism *A. hydrophila* is a common false positive in water quality monitoring methods that rely on the enzymatic testing of β‐galactosidase activity because it is of telluric rather than enteric origin and thus not considered a coliform (Waltman *et al*., 1982; Cabral, 2010). *A. hydrophila* was detected electrochemically with ResGal at 30 and 37°C (Fig. 3) and because it is reportedly electrogenic (Pham *et al*., 2003; Logan, 2009a,b), its ability to interfere with bioelectroanalytical TC enumeration was investigated in more detail to further support the notion of biological interference over abiotic environmental effects.

In a test with a constant inoculum size of *E. coli* (5.0 × 10^5^ CFU ml^−1^), but with an increasing *A. hydrophila* concentration from 5.0 × 10^2^ to 5.0 × 10^5^ CFU ml^−1^ (equivalent to 1000:1, 100:1, 10:1 and 1:1), a decrease in enumeration time from ~7.5 to ~4.7 h was observed, indicating that *A. hydrophila* strongly influences the detection signal in a binary culture of *E. coli* and *A. hydrophila* at 30°C (Fig. 3). Furthermore, enumeration time is likely to be dominated by *A. hydrophila* as the combined incubation is similar to that of a pure culture of *A hydrophila* at 30°C (Fig. 3), indicating suppression of *E. coli* by *A. hydrophila* or that its electrogenic character is dominant (Pham *et al*., 2003). Although the inability to differentiate coliforms, such as *Kleibsella* spp. and *Citrobacter* spp., is expected for galactosidase enzymatic tests, the inability to differentiate between *E. coli* and *A. hydrophila* means that this technique will overestimate *E. coli* or TCs at ambient temperatures in the presence of hydromonads. Interference from hydromonads is a well‐documented shortcoming of enzymatic β‐galactosidase detection and four commercially available methods exhibited this problem upon testing (Manafi, 2000). Hydromonad interference can be mitigated by the incorporation of selective inhibitors into the medium but is beyond the scope of this contribution (Geissler *et al*., 2000; Manafi, 2000). However, in principle, bioelectroanalytical TC quantification is possible and could be achieved at ambient tropical temperatures with shortcomings similar to what would be expected using established enzymatic β‐galactosidase detection methods and with only minor impact on enumeration times if an appropriate selective medium was incorporated into the tests.

### Applicability of bioelectroanalytical approach to TTC detection

**Figure 4:**
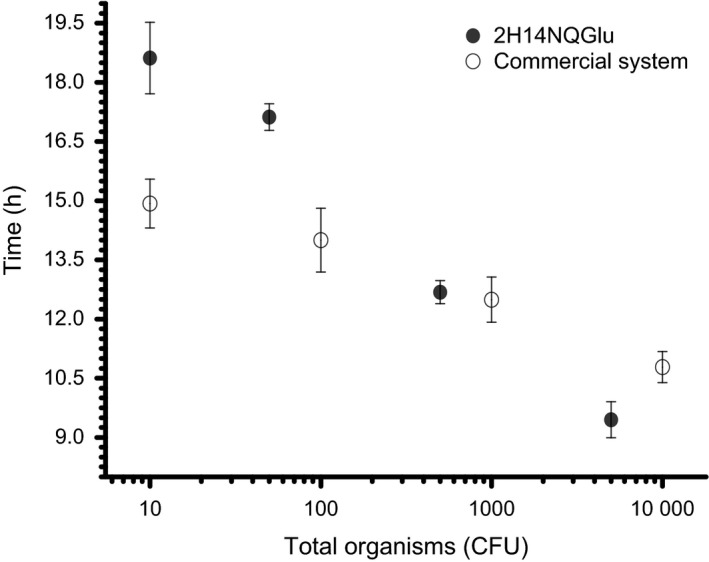
Detection time of *Escherichia coli* at 44.5°C with 2H14NQGlu at initial inoculum sizes of 10, 50, 500 and 5000 CFU (equivalent to 1, 5, 50 and 500 CFU ml^−1^) compared with a commercial detection system of 10, 100, 1000, and 1000 CFU (data from James *et al*., 2010). Error bars represent 1 standard deviation with *n* = 3 except for the commercial system where *n* = 5.

Enzymatic tests at 44.5°C are sometimes used to select for *E. coli*. It is preferably referred to as the TTC test because some *Shigella* spp. and *Salmonella* spp. also detect positive using this method. This apparent lack of specificity has its origins in the historical assignment of these organisms to different genera based on the pathology of the diseases they cause. However, with modern molecular insights, these taxa would be consigned to the same genus (Lan and Reeves, 2002). In a test with a constant inoculum size of *E. coli* (5.0 × 10^5^ CFU ml^−1^) but an increasing inoculum size of *A. hydrophila* (from 5.0 × 10^2^ to 5.0 × 10^5^ CFU ml^−1^, equivalent to 1000:1, 100:1, 10:1 and 1:1) and using 2H14NQGlu as a detection compound, the enumeration time remained reasonably constant (~2–2.9 h) which was not observed at 37°C, indicating that the contribution of *A. hydrophila* to the signal was minor at 44.5°C but had a significant impact at 37°C (Fig. 4). However, there was some attenuation of the enumeration time when the ratio of *A. hydrophila* to *E. coli* was high (0.1:1 and 1:1, Fig. 3) suggesting a competitive effect between *A. hydrophila* and *E. coli*. In a real situation, this would manifest as a slight overestimation of the total number of *E. coli* in the sample. As this effect is only apparent at both high inoculum density and when the relative concentration of *A. hydrophila* compared to *E. coli* is high (1:1), it would not pose significant problems in field applications, nor would the extent of interference be above that which would be expected with chromogenic β‐glucuronidase enzymatic tests. The electrochemical activity of *A. hydrophila*, thought to be achieved by direct electron transfer via c‐type cytochromes, was previously shown to be dependent on growth conditions, it is a noteworthy observation that there appears to be a temperature dependence too (Pham *et al*., 2003).

It has been demonstrated that the utility of the electrochemical glycosides is equivalent to their commercially available chromogenic counterparts. Nonetheless, it is clear that the presence of environmental organisms will have an effect on the accuracy of the enumeration times in field settings. This problem is shared by chromogenic, fluorogenic and bioelectroanalytical enzymatic tests alike, which rely on glycosides to detect the presence of β‐glucuronidase or β‐galactosidase. Interference by non‐target organisms is usually overcome by inclusion of a selective medium. The most common component of selective media for *Enterobacteriacea* is bile salts whose utility has been shown here. To completely suppress all non‐target organisms that interfere with enzymatic coliform or *E. coli* detection assays requires the incorporation of a mixture of specific inhibitors into the medium. Examples include antifungals such as amphotericin; rosolic acid to suppress organisms that grow at 44.5°C (e.g. *Staphylococcus aureus*) and cephalosporins such as cefsulodin that selectively inhibit Aeromonads (Presswood and Strong, 1978; Geissler *et al*., 2000; Manafi, 2000). The ideal medium for bioelectroanalytical detection of *E. coli* that suppresses non‐target fungi and bacteria should be the subject of further investigations.

### Performance against commercial system

Inoculum sizes in the range 5.0 × 10^2^–5.0 × 10^5^ CFU ml^−1^ are useful to demonstrate proof of principle, but we acknowledge that this is not an ideal range for commercial purposes. U.S. EPA drinking water standards recommended samples are free from *E. coli,* whereas recreational water quality standards recommend a threshold of 100 CFU *E. coli* in 100 ml of water with a statistical threshold value of 320 CFU in 100 ml, which must not be exceeded by 10% of samples taken (U.S. EPA, 2012). The market leading enzymatic tests achieve detection of 1 CFU *E. coli* in 100 ml in 12–48 h (Colifast, 2016, Idexx, 2016).

Bearing in mind that the set‐up described here has not been optimized, we decided to extend the range down to 1 CFU ml^−1^ to investigate the current limits of the technique. To benchmark our technique against commercially available system that has a different reactor volume, the absolute number of organisms in the sample rather than the inoculum density has been used. At 44.5°C, the enumeration time for 1 CFU ml^−1^ (10 CFU) of *E. coli* in an electrochemical system was ~19 h and progressed in inverse fashion to less than 10 h for an inoculum size of 500 CFU ml^−1^ (5000 CFU). However, at low inoculum density, the times are less reproducible (Fig. 4). Poor reproducibility at low inoculum density is to be expected as differences in inoculum size begin to disproportionately contribute to the error of the technique. With the small volume ECs, achieving commercial detection targets (0.01 CFU ml^−1^) is difficult as the average number of cells per reactor would be less than 1 CFU (effectively 0 CFU in 9 reactors of 10). To overcome this limitation would require larger volume ECs and the application of an iterative statistical approach, which is impractical with the current system. However, with some design modifications larger‐scale reactors could be developed to bring this method in line with commercial and regulatory standards.

A commercially available, U.S. EPA approved, automated *E. coli* detection system that uses a chromogenic method and a similar time‐to‐detection quantification procedure as used here can quantify 10 (0.1 CFU ml^−1^) organisms in around 15 h (James *et al*., 2010) compared to 19 h achieved with our system (Fig. 4). However, we achieved similar enumeration times of ~12.5 h for 500 (50 CFU ml^−1^) organisms and faster enumeration times of ~9 h for 5000 (500 CFU ml^−1^) organisms compared to 13.5 h for the commercial technique (James *et al*., 2010). Because of differences in reactor size and the precise conditions of the commercial technique (e.g. detection compound and concentration, and enumeration method) remain unknown, such a comparison is superficial but nonetheless illustrative. Whereas recognizing that additional optimization, medium development and validation is needed for the bioelectroanalytical approach, the results are very promising for an unoptimized system. Although the performance reported for the bioelectroanalytical detection of *E. coli* is not yet sufficient to assess drinking water quality, the scope for improved enumeration times and its current utility for environmental monitoring, subject to the inclusion of a selective medium, have been demonstrated.

## Conclusions

A bioelectroanalytical approach to *E. coli* enumeration using redox‐active glycosides has been demonstrated and the rational design of redox‐active glycosides, 2H14NQGal and 2H14NQGlu, led to both cost and performance improvements compared with commercially available compounds. The potential application of these novel glycosides in bioelectroanalytical detection of *E. coli* is feasible opening the door for further development towards alternative, low‐cost bioelectroanalytical detection systems. It is clear, however, that the development of a selective medium that hastens the recovery of environmentally stressed cells along with scale up to 100 ml volumes is a research priority which will allow validation of this method against U.S. EPA standards. We contend that bioelectroanalytical approaches offer much scope for future development including better performing glycosides, novel detection media and functionalized electrodes and that, potentially, a number of performance benefits such as more rapid detection in a better range of matrices could be realized. Importantly, the approach opens up a new research space in an area that has not, commercially at least, reflected the innovative trends apparent in research literature.

## Conflicts of interest

The authors declare no competing financial interest.
